# Effect of Sn and Ru in Pt-Based Catalysts for Alcohol Oxidation in Alkaline Media: A Combined Electrochemical and DFT Study

**DOI:** 10.3390/ma19143141

**Published:** 2026-07-22

**Authors:** Diego González-Quijano, Wilian Jesús Pech-Rodríguez, Eduardo Rubio, Gladis Guadalupe Suárez-Velázquez, Jesús Adrián Díaz-Real, Francisco Javier Rodríguez-Varela

**Affiliations:** 1 Departamento de Ingeniería Biomédica y Energías Renovables, Centro de Ciencias de la Ingeniería, Universidad Autónoma de Aguascalientes, Aguascalientes 20340, Aguascalientes, Mexico; rubioacademic@gmail.com; 2 Departamento de Maestría en Ingeniería, Universidad Politécnica de Victoria, Ciudad Victoria 87138, Tamaulipas, Mexico; wpechr@upv.edu.mx; 3 Departamento de Ingeniería en Energía, Universidad Politécnica de Altamira, Nuevo Libramiento Altamira Km. 3, Santa Amalia, Altamira 89602, Tamaulipas, Mexico; gsuarezv@upv.edu.mx; 4 Centro de Investigación y Desarrollo Tecnológico en Electroquímica, CIDETEQ, Parque Tecnológico, Querétaro S/N, Sanfandila, Pedro Escobedo 76703, Querétaro, Mexico; jdiaz@cideteq.mx; 5 Sustentabilidad de los Recursos Naturales y Energía, Cinvestav Unidad Saltillo, Av. Industria Metalúrgica 1062, Ramos Arizpe 25900, Coahuila, Mexico; javier.varela@cinvestav.edu.mx

**Keywords:** DAFC, ethanol oxidation reaction, ethylene glycol oxidation reaction, alkaline fuel cell, Pt-based catalyst, DFT study

## Abstract

Pt-Sn/C and Pt-Ru/C electrocatalysts were synthesized by a polyol method at nominal atomic ratios of 1:1, 2:1, and 3:1, and evaluated for the ethanol oxidation reaction (EOR) and ethylene glycol oxidation reaction (EGOR) in alkaline media. EDS confirmed compositions close to the nominal values, XRD evidenced fcc Pt-M alloy formation, and ADF-STEM revealed well-dispersed nanoparticles below 3 nm. Cyclic voltammetry showed that both Sn and Ru enhance activity relative to Pt/C: Pt-Sn_1:1_ delivered the highest forward current density in the Sn series (1486 mA mg^−1^_Pt_ for EOR; 2583 mA mg^−1^_Pt_ for EGOR), whereas Pt-Ru shifted the onset to more negative potentials (down to −539 mV vs. SHE for EOR), with Pt-Ru_3:1_ reaching 1858 (EOR) and 2434 mA mg^−1^_Pt_ (EGOR). Chronoamperometry revealed higher current retention during EGOR than EOR for all catalysts, indicating fewer poisoning intermediates from ethylene glycol. DFT calculations of CO adsorption on 1:1 and 3:1 model surfaces rationalize the distinct roles of the two metals: Sn excludes CO from Sn sites at both compositions and weakens the Pt-CO bond by 0.602 eV at 1:1, while Ru weakens it moderately yet binds CO strongly at both compositions; the bifunctional supply of OH_ads_ by Ru, inferred from the more negative onset potentials, accounts for its higher activity. PDOS analysis links these trends to distinct Pt d-band modifications.

## 1. Introduction

The development of efficient Direct Alcohol Fuel Cells (DAFCs) has emerged as a promising strategy for sustainable energy conversion, offering high energy density and the potential to utilize renewable, liquid fuels [1]. Among the most viable candidates, ethanol (EtOH) and ethylene glycol (EG) have attracted significant interest due to their abundance, ease of transport, and favorable thermodynamic properties [2]. However, the widespread implementation of DAFCs is limited by the slow kinetics of alcohol oxidation and the propensity of the electrocatalytic surfaces to be poisoned by strongly adsorbed carbonaceous intermediates, primarily CO_ads_ [3,4].

Platinum (Pt) is among the most active catalysts for the oxidation of small organic molecules such as alcohols [2,5,6]. However, the effectiveness of pure Pt is disadvantaged by its susceptibility to poisoning, which blocks active sites and leads to a rapid decline in catalytic activity [4,6,7,8]. The main challenge in the oxidation of these complex alcohols is the requirement for the cleavage of carbon-carbon (C-C) bonds, which determines whether the reaction follows a complete oxidation pathway toward CO_2_ (C_1_) or a partial oxidation pathway toward organic acids (C_2_); the inability to efficiently break these bonds often leads to the accumulation of CO species on the surface, further inhibiting the reaction [3,4]. Although alkaline media are known to enhance the activity of Pt-based catalysts compared to acidic environments, due to the facilitated adsorption of hydroxyl (OH^−^) species and their ability to enhance the stability and longevity of the fuel cell components, the complete oxidation of alcohols to CO_2_ remains a significant challenge [3,9,10]. To mitigate these issues, alloying Pt with secondary metals is a widely adopted strategy to modulate the electronic structure (electronic effect) and provide oxygenated species at lower potentials (bifunctional effect) [3,8,11,12].

Among the bimetallic systems, Pt-Sn and Pt-Ru catalysts have shown outstanding performance. In Pt-Sn systems, Sn promotes the ethanol oxidation reaction (EOR) by facilitating the removal of CO_ads_ via a bifunctional mechanism [11]. Recent studies suggest that the interaction between Pt and Sn is critical for the initial dehydrogenation steps of ethanol, specifically by enhancing the rate constant of dehydrogenation via the α-hydrogen path [5]. Theoretical studies suggest that Sn exhibits a positive ligand effect, providing a smoother potential energy surface for the initial oxidation steps compared to pure Pt, which significantly increases both activity and selectivity [13]. In Pt-Ru systems, Ru acts as an efficient source of OH_ads_ at lower potentials than Pt, which is particularly effective for both the EOR and the ethylene glycol oxidation reaction (EGOR) [14]. The catalytic efficiency of these alloys is not only dependent on the presence of the second metal but is also highly sensitive to the atomic ratio (Pt:M) and the degree of alloying, as the surface stoichiometry dictates the number of available active sites and the strength of the intermediate adsorption [15,16,17,18].

Despite the amount of electrochemical data, a fundamental gap remains in correlating the macroscopic activity of Pt-Sn and Pt-Ru catalysts in alkaline media with the underlying electronic structure that governs CO adsorption and oxygenated-species supply on each surface [15,19,20]. Establishing this structure–activity link requires a robust correlation between macroscopic kinetic observations and microscopic surface descriptors. Density Functional Theory (DFT) calculations are used as a powerful tool to evaluate the catalyst stability and predict catalytic properties, allowing for a deeper exploration of the energetic landscape, providing insights into the adsorption energies and the thermodynamic stability of the intermediates identified experimentally [20,21,22].

In this work, we investigate the effect of Sn and Ru incorporation on the electrocatalytic activity of Pt toward the oxidation of ethanol (EOR) and ethylene glycol (EGOR) on Pt-Sn/C and Pt-Ru/C electrocatalysts supported on Vulcan XC-72 carbon. The catalysts were synthesized via a controlled polyol method with varying atomic ratios (1:1, 2:1, and 3:1) to evaluate the effect of composition on their catalytic activity and CO poisoning tolerance in a 0.5 M KOH electrolyte. The structural and chemical properties of the materials were confirmed using X-ray diffraction (XRD) and energy-dispersive X-ray spectroscopy (EDS) and scanning transmission electron microscopy (STEM). Density Functional Theory (DFT) calculations using the PBE functional via Quantum ESPRESSO were employed to describe the CO adsorption energetics and d-band electronic structure of the model surfaces, providing a molecular-level rationale for the observed electrochemical trends and contributing to the design of more efficient and poison-resistant electrocatalysts for DAFCs. This study directly compares Pt-Sn/C and Pt-Ru/C under identical synthesis and testing conditions in alkaline media, linking their EOR and EGOR activity to the electronic-structure modifications of Pt through a unified DFT description based on CO adsorption energetics and d-band analysis.

## 2. Materials and Methods

### 2.1. Reactants and Gases

The metal precursors (H_2_PtCl_6_ ∙ 6H_2_O, SnCl_2_ ∙ 2H_2_O and RuCl_3_) were purchased from Sigma-Aldrich (St. Louis, MO, USA). Analytical grade chemicals, including ethanol (EtOH), ethylene glycol (EG), 2-propanol, NaOH, KOH, and H_2_SO_4_, were used as received for the synthesis of catalysts and electrochemical characterization procedures. Vulcan XC-72 carbon (Cabot Corporation, Boston, MA, USA) was employed as a catalyst support. Nafion^®^ (5% *v*/*v* solution, DuPont, Wilmington, DE, USA) was utilized for catalytic ink preparation. For the electrochemical tests, ultra-high purity N_2_ and CO were used at different stages to keep the atmosphere inside the electrochemical cell. All reagents were employed without further purification.

### 2.2. Synthesis of Pt-M/C Catalysts

Pt-M/C (M = Sn, Ru) catalysts with a total metal content of 20 wt% and Pt:M atomic ratios of 1:1, 2:1, and 3:1 were prepared via the polyol method, as previously reported [11,23,24]. The dispersing media consisted of ethylene glycol (EG) and ethanol (EtOH) in a 96:4 *v*/*v* ratio. Specifically, the Vulcan support was dispersed in 48 mL of EG, while Pt-M/C catalysts were subsequently prepared using H_2_PtCl_6_ ∙ 6H_2_O and the M precursor (SnCl_2_ ∙ 2H_2_O or RuCl_3_); each metal precursor (in 1 mL of EtOH) was separately sonicated for 30 min. Details regarding precursor quantities are summarized in Table 1. These solutions were then combined by adding the Pt and M precursors separately and dropwise, in that order, and the mixture was magnetically stirred for 1 h. Subsequently, 1 mol L^−1^ NaOH was added dropwise to reach pH 12, and the suspension was heated at 130 °C for 3 h under stirring. After cooling to room temperature over 3 h, the pH was adjusted to approximately 2 using 1 mol L^−1^ H_2_SO_4_ and stirred for an additional 3 h. Finally, the resulting suspension was filtered, washed with deionized water, and dried at ambient temperature inside a desiccator.

A Pt/C catalyst was prepared by an identical procedure, except that the Pt precursor and Vulcan were initially dispersed in 2 mL of H_2_O and 48 mL of EG, respectively, and then sonicated for 30 min, followed by magnetic stirring for 1 h. The pH adjustment, heating, washing, and drying steps were followed as described above for the Pt-M/C catalysts.

### 2.3. Physicochemical Characterization

A Philips X’Pert diffractometer (Malvern Panalytical, Almelo, The Netherlands) (Cu Kα λ = 1.54 Å) was employed to record XRD patterns between 10 and 100° (2θ). Structural parameters, including crystallite size (τ), lattice parameter (*a*), and alloying degree (M_alloy_) were determined from the XRD data according to the Scherrer, Bragg, and Vegard laws, respectively [25,26]. Surface chemical analysis was performed through energy-dispersive X-ray spectroscopy (EDS) using a Philips XL30 SEM operating at 20 kV (Philips Electron Optics, Eindhoven, The Netherlands). The morphology of the Pt-Sn_1:1_ and Pt-Ru_3:1_ samples was characterized by annular dark-field scanning transmission electron microscopy (ADF-STEM) using a JEOL JEM-ARM200F microscope operated at an accelerating voltage of 200 kV (JEOL Ltd., Tokyo, Japan).

### 2.4. Electrochemical Set-Up and Characterization

Electrochemical measurements were performed in a standard three-electrode cell using a VoltaLab PGZ 301 potentiostat/galvanostat (Radiometer Analytical (Hach Company), Lyon, France). The electrolyte was 0.5 M KOH solution saturated with N_2_. The working electrode consisted of a glassy carbon disk (5 mm diameter) onto which 10 µL of catalytic ink was deposited. The ink was prepared by dispersing 10 mg of catalyst powder in 1 mL of 2-propanol containing 5 µL of Nafion^®^ (5% *v*/*v* solution, DuPont, Wilmington, DE, USA), followed by sonication for 30 min to obtain a homogeneous suspension. A Pt foil served as the counter electrode, and a saturated Ag/AgCl (in KCl) electrode was used as the reference. All potentials reported in this work are referred to the standard hydrogen electrode (SHE). Measurements were conducted at a scan rate of 20 mV∙s^−1^.

Cyclic voltammograms (CVs) for electrochemical characterization were acquired in N_2_-saturated electrolyte. For Pt/C and Pt-Sn/C catalysts series, the potential window was set between −700 and 500 mV vs. SHE. For Pt-Ru/C catalysts series, however, the upper potential limit was restricted to 50 mV vs. SHE (−700 to 50 mV vs. SHE). This narrower window was chosen because, at potentials above this threshold, Ru undergoes irreversible surface oxidation [27] and dissolution followed by redeposition [28,29], processes that alter the surface composition and compromise the reproducibility of successive scans. The catalytic activity toward the ethanol oxidation reaction (EOR) and ethylene glycol oxidation reaction (EGOR) was evaluated by CVs recorded in the potential interval between −700 and 500 mV vs. SHE for all catalyst series. This broader range was necessary to capture the complete anodic oxidation profiles of the organic substrates, whose peak currents occur at potentials well above 50 mV vs. SHE. Under these conditions, the surface of Pt-Ru/C catalysts is exposed to higher anodic potentials; however, the presence of adsorbing reaction intermediates is known to modify the oxidation behavior of Ru compared to measurements in blank electrolyte [28].

Chronoamperometric measurements were performed for the EOR and EGOR by holding the electrode at a constant potential of −100 mV vs. SHE for 30 min in N_2_-saturated 0.5 M KOH containing 0.5 M EtOH or 0.5 M EG. The potential of −100 mV vs. SHE was selected as it lies within the electrochemically active region (above all onset potentials) while remaining below the maximum peak potentials for all catalysts, enabling a fair comparison of electrochemical stability under comparable operating conditions.

### 2.5. DFT Calculation Details

The Density Functional Theory (DFT) study was conducted using the Quantum ESPRESSO 7.5 software package. The methodology for the DFT calculations, including SCF, NSCF and PDOS analysis, was implemented following the procedure described in the Quantum ESPRESSO 7.0 Course for Solid-State Physics [30]. The exchange-correlation potential was described by the Generalized Gradient Approximation (GGA) with the Perdew-Burke-Ernzerhof (PBE) functional. Ion-electron interactions were modeled using the Standard Solid-State Pseudopotentials (SSSP) Efficiency library, specifically employing ultrasoft pseudopotentials.

To ensure accurate electronic structure calculations, an optimized plane-wave kinetic energy cutoff of 40 Ry was used. The Brillouin zone was sampled using a Monkhorst-Pack grid for the bulk system, while the surface slab model maintained a k-point spacing of 0.44 Å^−1^. The support effect was ignored to isolate the intrinsic catalytic properties of the Pt-M active site. All calculations were performed in vacuum; the alkaline electrolyte, solvation, and adsorbed hydroxyl (OH_ads_) species were not modeled explicitly. The DFT results are therefore used to explain the intrinsic, electronic-structure origin of the CO-surface interaction, while the bifunctional role of OH_ads_ is inferred from the electrochemical response rather than computed directly.

Structural optimizations were performed using the Broyden-Fletcher-Goldfarb-Shanno (BFGS) algorithm, with convergence criteria set to 10^−6^ Ry for total energy and 5 × 10^−4^ Ry/bohr for maximum force on any atom. The bulk alloy was fully relaxed using a variable-cell calculation (vc-relax).

Surface adsorption of carbon monoxide (CO) was investigated by placing the molecule on high-symmetry sites. The Pt-M (M = Sn, Ru) bimetallic surfaces were modeled as substitutional disordered alloys built on the Pt(111) fcc lattice. To maintain computational efficiency while capturing the essential electronic modifications induced by alloying, model surfaces with 1:1 and 3:1 stoichiometries were selected as representative for both systems.

Adsorption energy (E_ads_) was calculated using a four-layer slab within a periodic supercell. The bottom two layers were fixed, while the topmost layers and adsorbed molecules were allowed to relax. For each high-symmetry adsorption site, the CO molecule was initially positioned ~3 Å above the surface metal atom and subsequently relaxed to its equilibrium geometry using BFGS. The adsorption energies (E_ads_) were calculated according to Equation (1) [31,32]:

(1) $$E_{ads}=E_{\mathrm{slab}+\mathrm{CO}}-(E_{\mathrm{slab}}+E_{CO})$$

where E_slab+CO_, E_slab_, and E_CO_ represent the total energies of the Pt-M slab (M = Sn or Ru) with adsorbed CO, the clean slab, and the free CO molecule, respectively.

Projected density of states (PDOS) calculations were performed on the optimized slab models using a denser k-point mesh. The PDOS was projected onto the d-orbitals of surface Pt atoms and the second metal (M = Sn or Ru) to analyze contributions near the Fermi level. The d-band center (ε_d_) was obtained by numerical integration of the Pt-d band PDOS relative to the Fermi level.

## 3. Results and Discussion

### 3.1. Structural and Physicochemical Characterization

**Figure 1:**
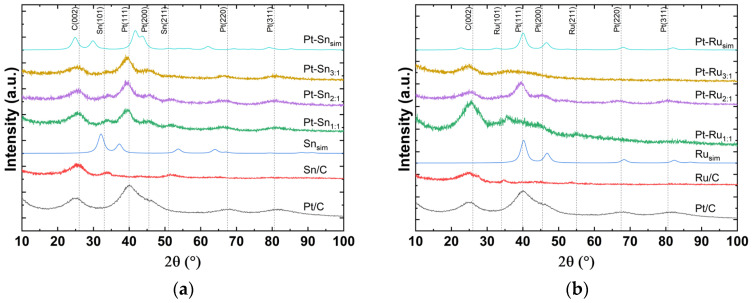
XRD patterns of synthesized (**a**) Pt/C, Sn/C vs. Pt-Sn/C series and simulated Sn, and Pt-Sn catalyst and (**b**) Pt/C, Ru/C vs. Pt-Ru/C series and simulated Ru, and Pt-Ru catalyst.

The crystallographic properties of the Pt/C and Pt-Sn/C catalysts are shown in the diffraction profiles in Figure 1a. All samples exhibit a characteristic reflection at approximately 26° (2θ), corresponding to the (002) plane of the Vulcan carbon support. The face-centered cubic (fcc) phase of Pt is clearly identified by the reflections at 40, 46, 67 and 81°, allocated to the (111), (200), (220), and (311) crystallographic planes, respectively, according to the Pt JCPDS 040802 card.

The diffractogram of the monometallic Sn/C reference is dominated by the carbon (002) reflection at 26° (2θ), with only weak and broad features at higher angles. The absence of the sharp reflections present in the simulated tin pattern (Sn_sim_) indicates that the supported Sn phase is poorly crystalline and highly dispersed on the carbon support, rather than forming well-defined metallic Sn crystallites.

For the Pt-Sn/C series, the emergence of peaks at 33 and 51° (2θ) suggests the presence of SnO_x_ species according to JCPDS 411445 card. The shift of the Pt(220) reflection toward lower diffraction angles is clearly observed compared to the monometallic Pt/C, which serves as strong evidence for the formation of a Pt-Sn alloy. Quantitative analysis, via the Vegard’s law and the Scherrer equation, indicates an estimated degree of alloying (M_alloy_) between 27.05% and 54.62%, with crystallite sizes (τ) ranging from 2.19 to 2.25 nm (Table 2). The observed expansion of the lattice parameter (*a*) further confirms the integration of Sn into the Pt matrix. These data suggest that neither the crystallite size (τ) nor the lattice parameter exhibits a linear dependence on the Sn atomic fraction.

Regarding the Pt-Ru/C catalysts (Figure 1b), the diffraction patterns are characterized by broad reflections, indicative of low crystallinity and highly dispersed active phases. The Ru/C reference likewise displays essentially only the carbon (002) reflection at 26° (2θ), without the resolved reflections of the simulated ruthenium pattern (Ru_sim_), confirming that the supported Ru is present as a highly dispersed, low-crystallinity phase.

While the Vulcan support peak remains constant at 26° (2θ), the Pt fcc reflections are only clearly observable in the Pt-Ru_2:1_ sample. For this specific catalyst, a degree of alloying of 42.26%, and a crystallite size of 1.23 nm were determined (Table 2). The Pt-Ru_1:1_ and Pt-Ru_3:1_ samples are not included in Table 2 because their diffraction patterns exhibit an amorphous profile due to a particle size below the XRD detection limit, as observed in previous studies [23], lacking sufficiently defined Pt fcc reflections to reliably apply the Scherrer, Bragg, and Vegard relationships. Accordingly, the structural parameters in Table 2 are reported only for Pt-Ru_2:1_, and no alloying-degree or structure-activity trends are inferred for the Pt-Ru/C series as a whole from the XRD data.

The observed divergences between the experimental patterns and the simulated models (Pt-M_sim_) are attributable to the inherent limitations of idealized crystallographic simulations. While simulations assume a perfect unit cell, the synthesized catalysts suffer lattice expansion because of the substitution of Pt by the Sn atom, shifting the peaks to lower 2θ values. Moreover, the experimental samples contain defects and internal strains caused by heat treatment that the ideal mathematical models usually ignore. This is confirmed by the broadening of the peaks in the data, which indicates that the crystals are nanometric, unlike simulations that represent perfectly stable states. Additionally, small instrumental offsets in the diffractometer may contribute to the slight positional shifts relative to the theoretical databases [33,34,35,36].

**Figure 2:**
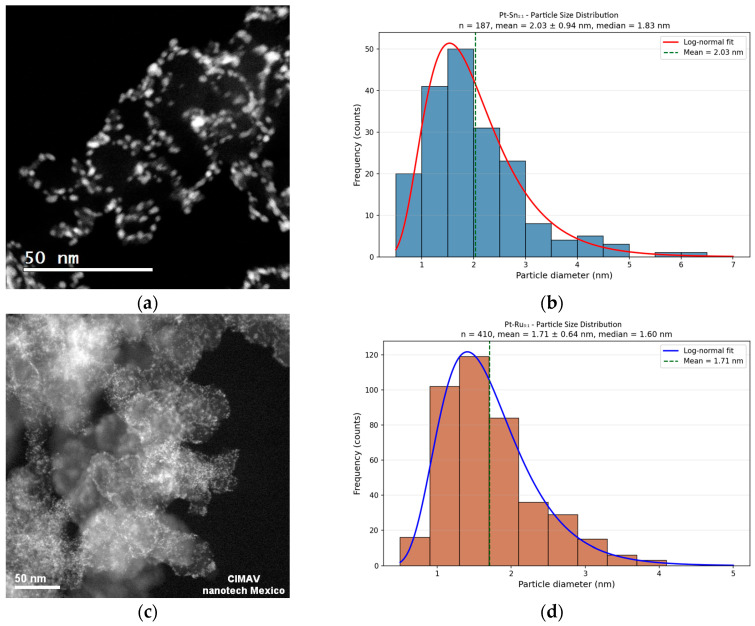
ADF-STEM micrographs and particle size distribution histograms of Pt-Sn_1:1_ (**a**,**b**) and Pt-Ru_3:1_ (**c**,**d**).

The ADF-STEM micrograph of Pt-Sn_1:1_ catalyst (Figure 2a,b) reveals metallic nanoparticles well dispersed across the carbon support. Quantitative analysis of individual particles yields a mean diameter of 2.03 ± 0.94 nm.

The distribution is positively skewed and is well described by a log-normal function, consistent with a nucleation-and-growth mechanism for supported metal nanoparticles. The small mean particle size and the comparatively narrow distribution indicate a high degree of metal dispersion, which provides a high electrochemically active surface area per unit mass of metal and is therefore favorable for electrocatalytic alcohol oxidation.

For Pt-Ru_3:1_ the ADF-STEM micrograph (Figure 2c,d) reveals metallic nanoparticles distributed over the carbon support. Quantitative analysis yields a mean diameter of 1.71 ± 0.64 nm. The very small mean particle size, together with the narrow distribution, indicates a high degree of metal dispersion on the support, which translates into a large electrochemically active surface area per unit mass of metal and is therefore favorable for electrocatalytic alcohol oxidation.

The Pt-Ru_3:1_ catalyst shows finer and more homogeneously sized particles, while the Pt-Sn_1:1_ system presents a somewhat broader distribution. The finer particle size of the Pt-Ru_3:1_ catalyst is consistent with the generally higher metal dispersion reported for Pt-Ru systems and is expected to contribute to a larger active surface area. The mean particle sizes are in good agreement with the crystallite sizes estimated from the XRD patterns in Table 2 for the Pt-Sn catalysts. For the Pt-Ru_3:1_ catalyst, however, the extremely small particle size prevents its detection by XRD: such fine particles broaden any diffraction peaks to the point where they merge with the background, so the material appears X-ray amorphous despite the well-dispersed metallic nanoparticles clearly resolved by transmission electron microscopy. This behavior reflects the detection limit of the technique under the available instrumental conditions. Overall, these results demonstrate that the employed synthesis route, based on the polyol method, effectively restricts particle growth to below 3 nm without requiring external stabilizing agents.

The elemental composition of the Pt/C, Pt-Sn/C, and Pt-Ru/C catalysts was determined via EDS, as summarized in Table 3. The carbon content across all samples remained consistently near the theoretical value of 80 wt%, confirming a uniform distribution of the support. For the Pt-Sn/C series, the experimentally determined Pt-Sn atomic ratios were 1:1, 1.6:1, and 2.4:1, which are in close agreement with the nominal compositions. Similarly, the Pt-Ru/C catalysts series exhibited stoichiometric ratios of 1:1, 1.8:1, and 3.2:1, respectively, showing closer agreement with the theoretical values. Additionally, the oxygen content was found to be between 3.07 and 5.01% for the Pt-Sn/C samples, whereas the Pt-Ru/C catalysts exhibited oxygen levels between 3.51 and 5.56%, indicating that the actual elemental composition of the catalysts is consistent with the calculated synthesis parameters.

### 3.2. Electrochemical Behavior and Performance

**Figure 3:**
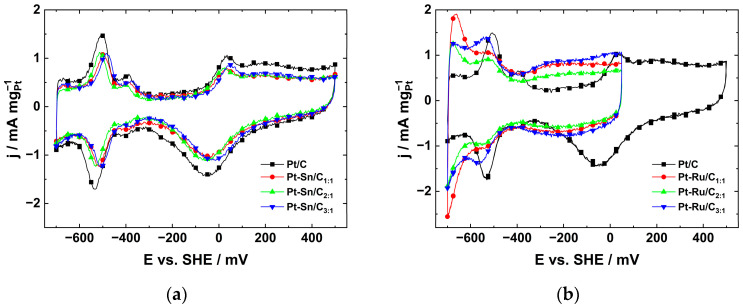
CVs of Pt/C vs. (**a**) Pt-Sn/C series and (**b**) Pt/C vs. Pt-Ru/C series. Electrolyte: N_2_-saturated 0.5 M KOH. Scan rate: 20 mV ∙ s^−1^.

The cyclic voltammograms (CVs) of the Pt/C, Pt-Sn/C and Pt-Ru/C catalysts series in alkaline media (N_2_-saturated at 0.5 M KOH) are presented in Figure 3.

All catalysts exhibit the hydrogen adsorption/desorption (H_ads_/H_des_) region and the double-layer region characteristic of platinum in alkaline media. For the Pt/C and Pt-Sn/C series, the PtO_x_ formation/reduction peaks are also observed. In the Pt-Ru/C series these peaks are absent, as the upper potential limit was restricted to 50 mV vs. SHE to prevent the irreversible surface oxidation and dissolution of Ru; this limit lies below the onset of PtO_x_ formation and therefore excludes those features from the accessible potential range.

The Pt-Sn/C catalysts series, Figure 3a, lead to a significant attenuation of the current density in the H_ads/_H_des_ regions compared to the monometallic Pt/C catalyst. This suppression of the hydrogen peaks is indicative of the electronic modification of the Pt surface (ligand effect) and the potential blocking of Pt active sites by Sn atoms [37,38].

The distinct behavior of the two series can be rationalized from the individual electrochemical signatures of Sn and Ru documented in alkaline media. Metallic Sn does not display H_ads_/H_des_ features; in alkaline electrolyte its voltammetric response is instead governed by surface oxidation to Sn(II) and Sn(IV) species [39,40]. Consequently, the incorporation of Sn attenuates the H_ads_/H_des_ charge and partially blocks the Pt sites, in agreement with the pronounced suppression of the hydrogen peaks observed in the Pt-Sn/C series. Ruthenium, in contrast, exhibits its own H_ads_/H_des_ together with surface redox and OH adsorption processes that develop within and adjacent to the hydrogen region [41]; this superposition accounts for the less pronounced attenuation of the H_ads_/H_des_ region in the Pt-Ru/C series and for the additional charge that contributes to the apparent ECSA values reported for this series (Section 3.2, Table 4).

The Electrochemically Active Surface Area (ECSA) values, calculated from the charge associated with the H_des_ region using the L_Pt_ and Q parameters in Equation (2), are summarized in Table 4. The same integration procedure was applied to the Pt/C, Pt-Sn/C, and Pt-Ru/C series, using the hydrogen desorption charge between approximately −700 and −400 mV vs. SHE. It should be noted, however, that for the Pt-Ru/C series this potential interval overlaps with the adsorption of oxygenated species and the surface redox transitions of Ru, which contribute additional charge that is not associated with hydrogen desorption. The ECSA values reported for the Pt-Ru/C series are therefore apparent values that overestimate the true hydrogen-derived active area and should be regarded as semiquantitative estimates rather than as directly comparable to those of the Pt/C and Pt-Sn/C series [42].

The Pt-Sn_1:1_ catalyst exhibited the highest ECSA (60.90 m^2^ g^−1^_Pt_), followed by Pt-Sn_3:1_ (52.63 m^2^ g^−1^_Pt_). Interestingly, the Pt-Sn_2:1_ sample showed an ECSA value (40.08 m^2^ g^−1^_Pt_) nearly identical to that of the Pt/C catalyst (42.65 m^2^ g^−1^_Pt_). The significant increase in ECSA for the Pt-Sn_1:1_ sample suggests a higher density of active sites available for the oxidation of fuel molecules.

(2) $$ECSA=\frac{Q}{210\;\mu C\;{cm}^{-2}\times L_{Pt}}$$

where

Q is the area under the curve in the H_des_ region, in μC cm^−2^.

L_Pt_ is the metallic Pt loading, in mg_Pt_ cm^−2^.

The conversion factor of 210 µC cm^−2^ corresponds to the charge associated with a full hydrogen monolayer on polycrystalline Pt [43]. For the Pt-Sn/C series this factor remains applicable because metallic Sn does not adsorb hydrogen in alkaline media [39,40], so the H_des_ charge originates from Pt sites; the site-blocking effect of Sn is reflected in the ECSA value itself. For the Pt-Ru/C series, the limitations discussed above apply and the values are reported as apparent estimates.

Figure 3b shows the CV profiles of the Pt/C and Pt-Ru/C catalysts. In this series, the alloyed catalysts show a less pronounced decrease in current density within the H_ads_/H_des_ region than those observed in the Pt-Sn/C series.

The calculated ECSA values for Pt-Ru_1:1_, Pt-Ru_2:1_, and Pt-Ru_3:1_ were 81.04, 53.09 and 74.68 m^2^ g^−1^_Pt_, respectively (Table 4). All Pt-Ru/C samples exhibit a higher apparent active area compared to Pt/C (42.65 m^2^ g^−1^_Pt_); even the Pt-Ru_2:1_ catalyst outperforms the ECSA of the monometallic platinum. The apparently high ECSA for Pt-Ru_1:1_ is at least partly associated with the larger contribution of Ru surface redox and OH adsorption processes to the integrated charge at the highest Ru content, and should therefore be interpreted with caution rather than as a purely morphological effect.

#### Catalytic Activity for the EOR and EGOR

**Figure 4:**
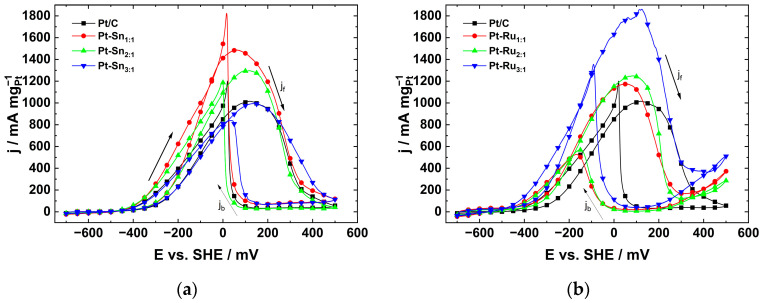
EOR at Pt/C vs. (**a**) Pt-Sn/C and (**b**) Pt-Ru/C catalysts series. Electrolyte: N_2_-saturated 0.5 M KOH + 0.5 M of C_2_H_5_OH. Scan rate: 20 mV ∙ s^−1^.

**Figure 5:**
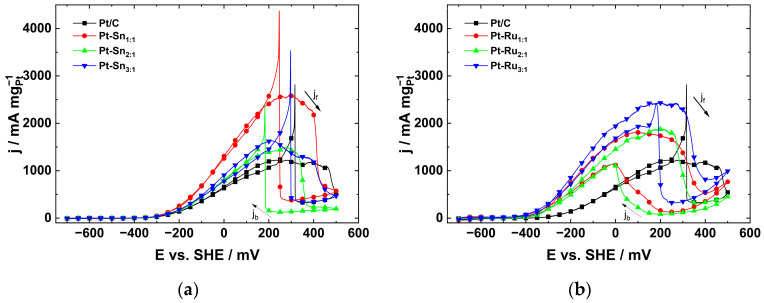
EGOR at Pt/C vs. (**a**) Pt-Sn/C and (**b**) Pt-Ru/C catalysts series. Electrolyte: N_2_-saturated 0.5 M KOH + 0.5 M of C_2_H_6_O_2_. Scan rate: 20 mV ∙ s^−1^.

The electrocatalytic performance of the Pt/C, Pt-Sn/C, and Pt-Ru/C catalysts for the ethanol oxidation reaction (EOR) and ethylene glycol (EGOR) was evaluated, as shown in Figure 4 and Figure 5, respectively. For the EOR process, the Pt-Sn_1:1_ catalyst (Figure 4a) exhibited a catalytic activity characterized by a maximum forward current density (j_f_) of 1486 mA mg^−1^_Pt_. Meanwhile, for Pt-Ru/C catalysts series (Figure 4b), the Pt-Ru_3:1_ delivered peak current densities of 1858 mA mg^−1^_Pt_. Both catalysts also exhibit a significant cathodic shift in the onset potential to −379 and −514 mV vs. SHE, respectively, compared to the Pt/C catalyst, which showed a lower current density of 1015 mA mg^−1^_Pt_ and a less negative onset potential of −336 mV vs. SHE.

The EGOR activity (Figure 5) followed a similar trend, with Pt-Sn_1:1_ and Pt-Ru_3:1_ emerging as the most efficient catalysts. These samples showed an onset potential of −285 and −440 mV vs. SHE and a peak current density of 2583 and 2434 mA mg^−1^_Pt_, respectively, representing an approximately double increase compared to the performance of Pt/C (Table 5). These results demonstrate that the incorporation of Sn or Ru as a co-catalyst significantly enhances the oxidation kinetics of both fuels. It is notable that Pt-Sn_1:1_ maintains superior performance despite its relatively low Pt content (1:1 atomic ratio), highlighting the synergistic effect between Pt and Sn in alkaline media. The Pt-Ru/C series exhibited a lower j_b_ (backward current) intensity, consistent with a reduced accumulation of carbonaceous intermediates on the catalyst surface, likely due to the bifunctional mechanism promoted by Ru, which provides oxygenated species (Ru-OH) that facilitate intermediate removal at lower potentials [44].

A comparative analysis of the onset potentials (Table 5) reveals that the Pt-Ru/C catalysts facilitate the oxidation reactions at significantly lower potentials than the Pt-Sn/C series. For instance, the Pt-Ru alloys exhibit onset potentials ranging from −489 to −539 mV vs. SHE for EOR and from −402 to −440 mV vs. SHE for EGOR, indicating a superior capability to start the reactions. This enhanced activity can be attributed to the bifunctional mechanism, where Ru promotes the formation of oxygenated species (OH_ads_) at lower potentials, thereby facilitating the removal of carbonaceous intermediates (such as CO_ads_) that typically poison the Pt surface.

The specific activities normalized to the real Pt surface area (j_spec_, Table 5) allow the intrinsic contribution to be separated from the surface-area effect. For EGOR, all bimetallic catalysts except Pt-Ru_1:1_ surpass Pt/C on a real-surface-area basis, confirming an intrinsic enhancement per active site. For EOR, Pt-Sn_2:1_ exhibits the highest specific activity (3.22 mA cm^−2^), whereas the j_spec_ of Pt-Sn_1:1_ (2.44 mA cm^−2^) is comparable to that of Pt/C (2.38 mA cm^−2^), indicating that its superior mass activity stems mainly from its larger ECSA. The benefit of the second metal for EOR is therefore expressed primarily through the more negative onset potentials and the increased active area, rather than through a higher intrinsic rate per site. For the Pt-Ru/C series, the j_spec_ values are lower-bound estimates, since the apparent ECSA overestimates the active area; even so, Pt-Ru_3:1_ matches or exceeds the specific activity of Pt/C.

**Figure 6:**
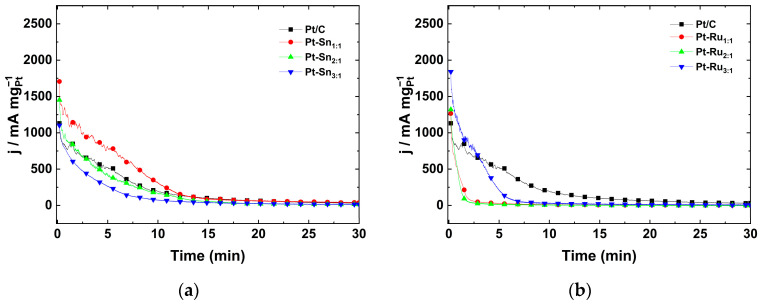
Chronoamperometric curves of EOR at Pt/C vs. (**a**) Pt-Sn/C and (**b**) Pt-Ru/C catalysts series. Electrolyte: N_2_-saturated 0.5 M KOH + 0.5 M of C_2_H_5_OH. Static potential: −100 mV vs. SHE.

Chronoamperometric measurements reveal distinct stability patterns among the catalyst series during EOR (Figure 6). Pt/C shows rapid current decay from initial current density (I_s_) of 1130.95 mA mg^−1^_Pt_ to final current density (I_f_) after 30 min of 30.38 mA mg^−1^_Pt_, while Pt-Sn/C catalysts (Figure 6a) display a I_s_ of 1705.79 mA mg^−1^_Pt_ (Pt-Sn_1:1_), 1451.06 mA mg^−1^_Pt_ (Pt-Sn_2:1_), and 1102.87 mA mg^−1^_Pt_ (Pt-Sn_3:1_) with faster current decay behavior compared to Pt/C (Table 6).

For the Pt-Ru/C series (Figure 6b), Pt-Ru_1:1_ and Pt-Ru_2:1_ show initial currents (I_s_) comparable to that of the Pt/C catalyst (1130.95 mA mg^−1^_Pt_), whereas Pt-Ru_3:1_ exhibits a higher initial current (1840.49 mA mg^−1^_Pt_). However, Pt-Ru_1:1_ and Pt-Ru_2:1_ exhibit a faster current decay compared to Pt/C and Pt-Ru_3:1_. This suggests that high Ru loading suppresses the sustained activity at −100 mV vs. SHE, while the composition with lower Ru content (Pt-Ru_3:1_) partially retains performance, indicating an optimal Pt:Ru ratio for stability under constant-potential conditions.

**Figure 7:**
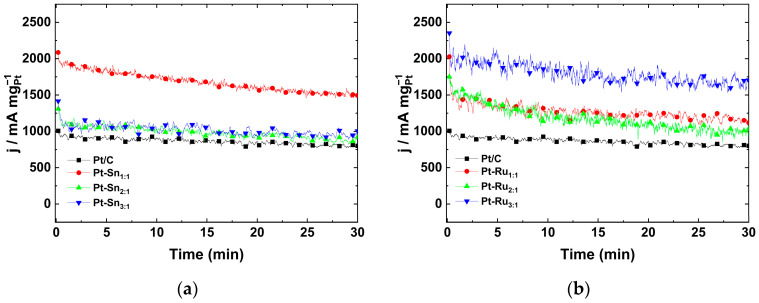
Chronoamperometric curves of EGOR at Pt/C vs. (**a**) Pt-Sn/C and (**b**) Pt-Ru/C catalysts series. Electrolyte: N_2_-saturated 0.5 M KOH + 0.5 M of C_2_H_6_O_2_. Static potential: −100 mV vs. SHE.

Chronoamperometric measurements during EGOR (Figure 7) at −100 mV vs. SHE reveal markedly superior current retention compared to EOR (Figure 6), indicating that ethylene glycol produces less poisoning surface intermediates.

Pt-Sn/C catalysts exhibit excellent stability for EGOR, while Pt-Sn_1:1_ and Pt-Sn_2:1_ showed a more stable behavior with an I_s_ of 2085.61 and 1308.06 mA mg^−1^_Pt_, respectively. Despite moderate EOR performance, this exceptional EGOR stability highlights a substrate-dependent poisoning behavior. Pt-Sn_3:1_ maintains comparable behavior but with lower current along the test, demonstrating that the Sn promoter effectively suppresses intermediate accumulation regardless of composition. Likewise, Pt-Ru/C catalysts series show I_s_ of 2028.03 mA mg^−1^_Pt_ (Pt-Ru_1:1_), 1748.79 mA mg^−1^_Pt_ (Pt-Ru_2:1_), and 2352.04 mA mg^−1^_Pt_ (Pt-Ru_3:1_) superior to their EOR performance.

The electrochemical results demonstrate that Sn and Ru enhance activity relative to Pt/C through distinct mechanisms: Pt-Sn_1:1_ achieves a peak current density of 1486 mA mg^−1^_Pt_ at 65 mV vs. SHE for EOR and the highest EGOR current density of the study (2583 mA mg^−1^_Pt_ at 296 mV vs. SHE), while Pt-Ru_3:1_ exhibits the highest EOR peak current density of the study (1858 mA mg^−1^_Pt_ at 121 mV vs. SHE) and the highest EGOR current density within the Pt-Ru series (2434 mA mg^−1^_Pt_ at 199 mV vs. SHE), and the most negative onset potential was observed for Pt-Ru_1:1_ (−539 mV vs. SHE) for EOR, indicating a lower thermodynamic barrier for the initiation of the reaction in this composition. During EGOR the materials sustained high current retention, indicating greater resistance to poisoning under prolonged operation; in contrast, during EOR all catalysts underwent severe current decay, with the bimetallic series retaining final currents comparable to or below that of Pt/C.

The shape of the chronoamperometric curves is governed by kinetic and surface-adsorption processes rather than by mass transport. At the applied potential of −100 mV vs. SHE, which lies well above the onset potentials of all catalysts (−282 to −539 mV vs. SHE, Table 5) but below their peak potentials, the reaction proceeds under kinetic/surface control; the reactant is not depleted from the bulk, so the current does not exhibit the sharp Cottrell-type decay characteristic of mass-transport limitation. Instead, the gradual current decay reflects the progressive accumulation of strongly adsorbed carbonaceous intermediates (CO_ads_ and related species) that lower the fraction of available active sites over time. The markedly slower decay and higher current retention observed during EGOR compared with EOR (Figure 6 and Figure 7) are therefore consistent with a lower steady-state coverage of poisoning intermediates from ethylene glycol. This behavior also reconciles the apparent contrast between the cyclic voltammetry and chronoamperometry results: the incorporation of Sn or Ru increases the forward peak current density in the potential sweep, reflecting faster oxidation kinetics, but does not by itself prevent the progressive CO poisoning that governs the steady-state current under prolonged constant-potential operation. Consequently, the enhancement provided by the second metal is most clearly expressed as higher instantaneous activity in cyclic voltammetry rather than as improved long-term retention in chronoamperometry, particularly for the ethanol-derived intermediates. The identity of the adsorbed intermediates is not determined experimentally in this work; the interpretation is therefore restricted to the CO adsorption energetics computed by DFT and the current retention observed electrochemically.

### 3.3. Density Functional Theory (DFT) Calculations

**Figure 8:**
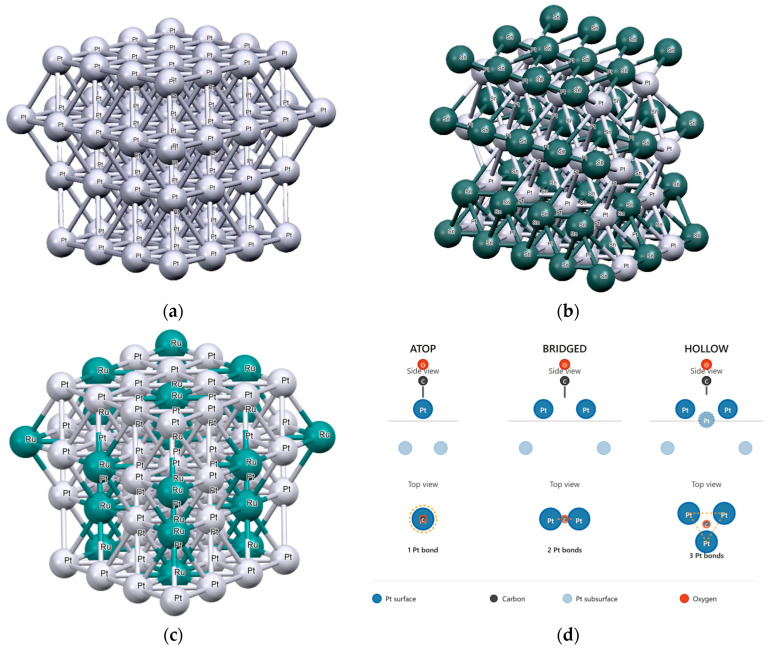
View of the optimized models used in the DFT calculations: (**a**) Pt(111), (**b**) Pt-Sn_1:1_, and (**c**) Pt-Ru_3:1_, and (**d**) view of different surface sites for CO adsorbed at the atop, bridged, and hollow sites.

To establish a molecular-level understanding of this divergent behavior, DFT-calculated CO adsorption energies on Pt(111), and on Pt-Sn and Pt-Ru model surfaces at 1:1 and 3:1 stoichiometries were correlated with the electrochemical findings. CO adsorption was evaluated at high-symmetry surface sites (atop, bridged, and hollow). Figure 8 presents the optimized models for Pt(111) (a), Pt-Sn_1:1_ (b), and Pt-Ru_3:1_ (c), corresponding to the most active composition of each bimetallic series (Table 5), and a schematic representation of the adsorption sites (d).

The FCC(111) surface contains three high-symmetry adsorption sites. The atop (or on-top) site is where the adsorbate molecule sits directly above a single surface Pt atom, forming a single Pt-C bond with a coordination number of 1. The bridged (or bridge) site occurs when an adsorbate positions itself between two adjacent surface Pt atoms, forming two equivalent Pt-C bonds with a coordination number of 2. The hollow site (3-fold or fcc-hollow) is where the adsorbate molecule sits within a triangular cavity formed by three nearest-neighbor surface Pt atoms, each contributing a Pt-C bond with a coordination number of 3. These three sites differ in their geometric arrangement and the number of metal atoms directly coordinating the adsorbate.

Since CO_ads_ is the primary poisoning intermediate for both ethanol and ethylene glycol oxidation in alkaline media, its adsorption energy serves as a primary descriptor for the poison-tolerance of these catalysts.

Theoretical simulations reveal that the incorporation of Ru modulates the d-band center of Pt, leading to a weaker CO binding energy on Pt sites compared to monometallic Pt. The early generation of OH_ads_ species at low potentials, although not modeled explicitly here, is inferred from the markedly more negative onset potentials measured for the Pt-Ru series. Together, the weakened CO-Pt interaction and the experimentally evidenced early OH supply account for the continuous oxidation of carbonaceous residues during the forward scan, so that the surface retains a high fraction of active sites, consistent with the lower overpotential and the reduced j_b_ peak intensity observed in the voltammograms.

Although metallic Ru adopts an hcp structure in its bulk form, the Pt-Ru_2:1_ sample exhibits fcc reflections by XRD (Figure 1b and Table 2), consistent with the formation of a Pt-based fcc solid solution [45]. This fcc host lattice is expected for Pt-rich compositions and is consistent with the well-dispersed metallic nanoparticles evidenced by ADF-STEM for Pt-Ru_3:1_ (Figure 2). Accordingly, the Pt-Ru bimetallic surfaces were modeled on the Pt(111) fcc lattice, with Pt atoms substituted by Ru at fractions corresponding to the experimental 1:1 and 3:1 atomic ratios. The same disordered substitutional approach was used for Pt-Sn. Following the convention of Equation (1), more negative E_ads_ values indicate stronger CO adsorption (Table 7).

On Pt_sim_, the calculated E_ads_ values follow the order: hollow > top > bridge (E_ads_ = −1.876, −1.735, −1.636 eV, respectively), consistent with the documented PBE site-preference inversion for the CO/Pt_sim_ system [45,46]. For the alloys, distinct modifications emerge:

(a) On Pt-Sn_1:1sim_, the CO-Pt interaction is weakened by 0.602 eV (from −1.735 to −1.133 eV) through the electronic (ligand) effect of Sn. More notably, CO does not bind on Sn_Atop_ (0.016 eV) nor on mixed three-fold hollow sites (−0.052 eV), restricting the CO adsorption exclusively to Pt-only configurations.

(b) On Pt-Ru_1:1sim_, the ligand effect is also observed but is weaker (0.361 eV weakening on Pt_Atop_). However, the key difference is that Ru itself binds CO strongly (E_ads_ at Ru_Atop_ = −2.131 eV); in contrast to Pt-Sn_1:1sim_, CO adsorption on Pt-Ru_1:1sim_ is not site-restricted: both Pt and Ru atoms accommodate CO_ads_. The strong Ru-CO bond ensures that CO_ads_ species persist long enough to be oxidized by OH_ads_ generated on Ru sites through the bifunctional mechanism, which is operative at low potentials.

(c) On Pt-Sn_3:1sim_, the trend reverses only in its electronic component: the Pt-CO interaction is strengthened by 0.585 eV (from −1.735 to −2.320 eV), while Sn sites remain excluded from CO chemisorption: in the final relaxed geometry of the Sn_Atop_ calculation and bridged calculations, the molecule does not form a Sn-C bond and remains close to the surface, so the values of −0.916 eV and −0.924 eV reflect the interaction of these unbound configurations with the surrounding surface rather than chemisorption minima.

(d) On Pt-Ru_3:1sim_, the qualitative behavior found at the 1:1 composition is preserved: the Pt-CO bond is weakened by 0.153 eV at the Pt_Atop_ site (from −1.735 to −1.582 eV), while Ru retains a strong CO affinity (E_ads_ at Ru_Atop_ = −2.179 eV, essentially unchanged with respect to −2.131 eV at the 1:1 composition). The bridged site also binds CO strongly (−2.090 eV), whereas adsorption at the hollow site is weakened relative to Pt_sim_ (−1.507 vs. −1.876 eV). Across both compositions, therefore, the Pt-Ru surfaces combine a moderate electronic weakening of the Pt-CO interaction with strong CO retention on Ru sites; the enhanced activity of the Pt-Ru series is accordingly expressed through the early supply of OH_ads_ inferred from its more negative onset potentials (Table 5), rather than through a generalized destabilization of CO_ads_.

#### Electronic Properties Analysis

The d-band centers of the surface Pt_sim_ atom in each system, obtained by integration of the Pt-d PDOS, are summarized in Table 8. Both bimetallic surfaces exhibit a Pt-d band center more negative than monometallic Pt_sim_ (ε_d_ = −2.013 eV), confirming the operation of the electronic (ligand) effect introduced by the second metal. Notably, the magnitude of the d-band downshift does not follow the same order as the weakening of the Pt-CO bond: while Pt-Ru_1:1sim_ displays the larger ε_d_ downshift (Δε_d_ = −1.220 eV), it produces the smaller CO-Pt weakening (ΔE_ads_ = 0.361 eV); on the other hand, Pt-Sn_1:1sim_ shows a smaller d-band downshift (Δε_d_ = −0.392 eV) but a larger CO-Pt weakening (ΔE_ads_ = 0.602 eV).

The 3:1 compositions reinforce this non-linear picture: Pt-Sn_3:1sim_ combines an almost unshifted d-band center (Δε_d_ = −0.065 eV) with the strongest CO adsorption of the study (ΔE_ads_ = −0.585 eV), whereas Pt-Ru_3:1sim_ couples a moderate downshift (Δε_d_ = −0.305 eV) with a correspondingly moderate weakening (ΔE_ads_ = 0.153 eV at Pt_Atop_). Within the Pt-Ru series, the magnitude of the Pt-CO weakening thus follows that of the d-band downshift (0.361 eV for Δε_d_ = −1.220 eV at 1:1 versus 0.153 eV for Δε_d_ = −0.305 eV at 3:1), so the departure from the conventional d-band correlation is confined to the Pt-Sn series. In the case of Pt-Sn_3:1sim_, the strengthening of CO adsorption despite a nearly unchanged ε_d_ indicates that descriptors beyond the d-band center position, such as the band shape discussed below and local geometric contributions of the diluted Sn atoms, govern the CO-surface interaction at this composition.

**Figure 9:**
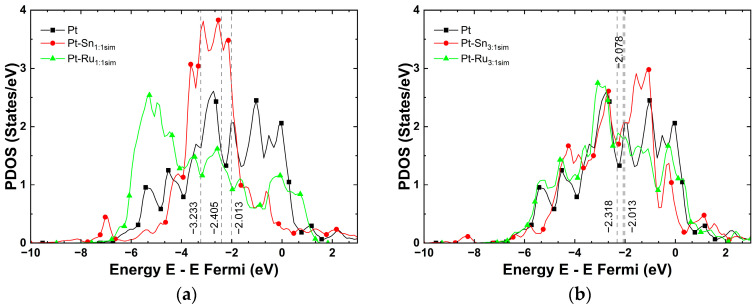
Projected density of states (PDOS) of the d-orbitals of the surface (111) Pt atom in (**a**) Pt_sim_ (black, ε_d_ = −2.013 eV), Pt-Sn_1:1sim_ (red, ε_d_ = −2.405 eV), and Pt-Ru_1:1sim_ (green, ε_d_ = −3.233 eV), and (**b**) Pt_sim_ (black, ε_d_ = −2.013 eV), Pt-Sn_3:1sim_ (red, ε_d_ = −2.078 eV), and Pt-Ru_3:1sim_ (green, ε_d_ = −2.318 eV) model surfaces. The Fermi level is set to zero (vertical dashed line). Numerical values of ε_d_ are marked.

The extended d-band model explains this non-linear correlation [47], which incorporates not only the position of the d-band center but also the bandwidth. The PDOS profiles (Figure 9a) reveal qualitatively different electronic responses to alloying: in Pt-Sn, the absence of accessible near-Fermi d-states on Sn prevents Pt-d/M-d hybridization, leading to a pronounced narrowing of the Pt-d band that is highly localized around −3 eV. In Pt-Ru_1:1sim_, in contrast, active Pt-d/Ru-d hybridization preserves a broad Pt-d band with significant density of states near the Fermi level. The narrow Pt-d band in Pt-Sn_1:1sim_, despite a smaller ε_d_ shift, highly limits the states available to hybridize with the CO 2π* orbital, resulting in a stronger Pt-CO weakening. The broad Pt-d band in Pt-Ru_1:1sim_, despite a larger ε_d_ shift, retains enough near-Fermi density to partially preserve the Pt-CO interaction.

The PDOS of the 3:1 surfaces (Figure 9b) is consistent with this framework. Upon dilution of Sn, the Pt-d band of Pt-Sn_3:1sim_ recovers a broad, Pt-like profile with substantial density of states near the Fermi level, in contrast to the pronounced narrowing observed at the 1:1 composition; this restored near-Fermi density preserves the states available to hybridize with the CO 2π* orbital and is consistent with the strengthened Pt-CO interaction despite a nearly unchanged ε_d_. For Pt-Ru_3:1sim_, the Pt-d band remains broad and moderately downshifted, an attenuated version of the behavior found at 1:1, in line with the correspondingly moderate Pt-CO weakening (0.153 eV) and with the conventional d-band correlation followed by the Pt-Ru series.

Together, these electronic descriptors provide a consistent electronic explanation for the catalytic behavior observed in the electrochemical measurements: on Pt-Sn_1:1sim_, the combination of strong Pt-CO weakening and the exclusion of CO from Sn sites (Table 7) is reflected in the more negative onset potential relative to Pt/C.

On Pt-Ru_1:1sim_, the moderate Pt-CO weakening combined with strong CO adsorption on Ru atoms (E_ads_ = −2.131 eV) is consistent with an enhanced tolerance to CO poisoning, in agreement with the lowest onset potential (−539 mV vs. SHE, observed for Pt-Ru_1:1_) and the highest peak current densities, measured for the Pt-Ru_3:1_ composition.

The 3:1 compositions extend this correlation in opposite directions for the two series. On Pt-Sn_3:1sim_, the strengthened Pt-CO interaction (−2.320 eV) suppresses the electronic advantage identified at the 1:1 composition, although Sn sites remain excluded from CO chemisorption, consistent with Pt-Sn_3:1_ delivering the lowest EOR peak current density of the study (993 mA mg^−1^_Pt_, Table 5). On Pt-Ru_3:1sim_, in contrast, the moderate Pt-CO weakening and the strong CO retention on Ru sites (−2.179 eV) persist at the lower Ru content, indicating that the descriptors associated with CO tolerance in the Pt-Ru series are robust across the composition range; this is consistent with the uniformly negative onset potentials of the series (−489 to −539 mV vs. SHE for EOR) and with the highest peak current densities measured for Pt-Ru_3:1_.

## 4. Conclusions

This work combined electrochemical measurements and DFT calculations to rationalize the distinct electrochemical behavior of ethanol and ethylene glycol oxidation in alkaline media on Pt/C, Pt-Sn/C and Pt-Ru/C catalysts with different atomic ratios. The polyol synthesis yielded well-dispersed nanoparticles below 3 nm, with mean diameters of 2.03 ± 0.94 nm for Pt-Sn_1:1_ and 1.71 ± 0.64 nm for Pt-Ru_3:1_, in agreement with the crystallite sizes estimated by XRD for the Pt-Sn/C series; Pt-Ru_3:1_ remains X-ray amorphous owing to its extremely small particle size. Both bimetallic systems enhanced the catalytic activity relative to Pt/C by lowering the onset potential and increasing the peak current density. For EOR, optimal performance was observed for Pt-Sn_1:1_ (1486 mA mg^−1^_Pt_ at 65 mV vs. SHE) and Pt-Ru_3:1_ (1858 mA mg^−1^_Pt_ at 121 mV vs. SHE). For the ethylene glycol oxidation reaction, both bimetallic systems similarly outperformed Pt/C, with Pt-Sn_1:1_ achieving 2583 mA mg^−1^_Pt_ at 296 mV vs. SHE and Pt-Ru_3:1_ reaching 2434 mA mg^−1^_Pt_ at 199 mV vs. SHE, alongside more negative onset potentials.

Chronoamperometry at −100 mV vs. SHE revealed a strong substrate dependence for all catalysts. During EOR the current decayed rapidly, with very low final current densities for both bimetallic series (13.49–35.99 mA mg^−1^_Pt_ for the Pt-Sn/C series and 0.71–7.76 mA mg^−1^_Pt_ for the Pt-Ru/C series, versus 30.38 mA mg^−1^_Pt_ for Pt/C), reflecting severe surface poisoning by ethanol-derived intermediates. During EGOR, in contrast, all catalysts sustained markedly higher currents (final values of 891.43–1496.33 mA mg^−1^_Pt_ for the Pt-Sn/C series and 959.71–1663.40 mA mg^−1^_Pt_ for Pt-Ru/C series), with Pt-Ru_3:1_ retaining the highest current (I_s_ = 2352.04, I_f_ = 1663.40 mA mg^−1^_Pt_). For the Pt-Ru/C series the stability under constant potential was composition-dependent, with Pt-Ru_3:1_ outperforming the more Ru-rich compositions. These results indicate that ethylene glycol generates fewer poisoning intermediates than ethanol. Under EGOR, both Sn and Ru markedly improved the current retention relative to Pt/C, whereas under EOR the final current densities of the bimetallic catalysts were comparable to or lower than that of Pt/C, indicating that the beneficial effect of the second metal on poisoning resistance is strongly substrate-dependent and is only evident for ethylene glycol.

DFT calculations provided an electronic-level explanation of these trends. The two bimetallic systems mitigate CO_ads_ poisoning through fundamentally different routes. Pt-Sn operates by site-exclusion and a composition-dependent electronic effect: CO does not chemisorb on Sn sites at either composition (E_ads_ = 0.016 eV on Sn_Atop_ at 1:1; unbound configurations at 3:1), restricting adsorption exclusively to Pt sites, while the Pt-CO bond is weakened by 0.602 eV at the 1:1 composition but strengthened at 3:1. Pt-Ru, in contrast, combines a moderate Pt-CO weakening with strong CO retention on Ru sites across both compositions (E_ads_ = −2.131 and −2.179 eV at Ru_Atop_); the bifunctional supply of OH_ads_, not modeled explicitly in this work, is inferred from the markedly more negative onset potentials of the series. Projected density of states analysis, interpreted within the extended d-band model, attributed these differences to qualitatively distinct modifications of the Pt-d band: a pronounced narrowing in Pt-Sn_1:1sim_ versus a broad downshift in Pt-Ru_1:1sim_, with the strengthened CO adsorption on Pt-Sn_3:1sim_ occurring despite a nearly unchanged d-band center. Ultimately, these electronic and bifunctional descriptors account for the distinct CO-tolerance behavior of Pt-bimetallic formulations.

Although Pt-Sn and Pt-Ru are well-established systems for alcohol oxidation, the present work advances their understanding by providing a unified experimental-theoretical explanation that links their different electrochemical behavior to distinct electronic-structure modifications of Pt.

These findings provide an electronic framework for the rational design of Pt-based bimetallic electrocatalysts: Sn enhances activity through site-blocking and electronic localization, while Ru combines the inferred bifunctional OH supply with active CO retention on Ru sites across the composition range.

## Figures and Tables

**Table 1:** Synthesis parameters and precursor ratios for Pt-Sn/C and Pt-Ru/C catalysts series.

Sample	Catalysts Elements	Nominal Pt:M (at. Ratio)	H_2_PtCl_6_∙6H_2_O (mg)	SnCl_2_∙2H_2_O (mg)	RuCl_3_ (mg)
Pt-Sn_1:1_	Pt, Sn, C	1:1	77.63	55.32	-
Pt-Sn_2:1_		2:1	95.74	34.11	-
Pt-Sn_3:1_		3:1	103.81	24.66	-
Pt-Ru_1:1_	Pt, Ru, C	1:1	69.97	-	28.02
Pt-Ru_2:1_		2:1	84.37	-	16.90
Pt-Ru_3:1_		3:1	90.59	-	12.09

**Table 2:** Structural characteristics of Pt/C, the Pt-Sn/C series, and Pt-Ru_2:1_ catalyst.

Sample	τ (nm)	M_alloy_ (%)	*a* (Å)
Pt/C	1.90	-	3.910
Pt-Sn_1:1_	2.19	30.14	3.994
Pt-Sn_2:1_	2.25	27.05	3.965
Pt-Sn_3:1_	2.25	54.62	3.978
Pt-Ru_2:1_	1.23	42.26	3.981

**Table 3:** Chemical characteristics of Pt/C, Pt-Sn/C, and Pt-Ru/C catalysts series.

Sample	Chemical Composition, EDS (wt%)					Pt:M Ratio, Nominal (at. Ratio)	Pt:M Ratio, EDS (at. Ratio)
	Pt	Sn	Ru	C	O_x_		
Pt/C	19.20	-	-	80.80	-	-	-
Pt-Sn_1:1_	8.60	5.18	-	81.21	5.01	1:1	1:1
Pt-Sn_2:1_	14.63	5.69	-	76.61	3.07	2:1	1.6:1
Pt-Sn_3:1_	12.14	3.02	-	80.51	4.33	3:1	2.4:1
Pt-Ru_1:1_	8.76	-	4.46	81.35	5.43	1:1	1:1
Pt-Ru_2:1_	10.26	-	2.99	83.24	3.51	2:1	1.8:1
Pt-Ru_3:1_	8.60	-	1.38	84.46	5.56	3:1	3.2:1

**Table 4:** Pt loading and electrochemical parameters of Pt/C, Pt-Sn/C, and Pt-Ru/C catalysts series in alkaline media.

Sample	Electrode_Pt Loading_ (mg Pt)	Q (µC cm^−2^)	ECSA (m^2^ g^−1^_Pt_)
Pt/C	0.0193	8.82	42.65
Pt-Sn_1:1_	0.0091	5.94	60.90
Pt-Sn_2:1_	0.0153	6.59	40.08
Pt-Sn_3:1_	0.0125	7.05	52.63
Pt-Ru_1:1_	0.0087	7.60	81.04
Pt-Ru_2:1_	0.0110	6.30	53.09
Pt-Ru_3:1_	0.0088	7.08	74.68

**Table 5:** Electrocatalytic parameters of the EOR and EGOR at Pt/C, Pt-Sn/C, and Pt-Ru/C catalysts series in alkaline media.

Sample	EOR			EGOR		
	Onset Potential (mV)	j_f_ (mA mg^−1^_Pt_)	j_spec_ (mA cm^−2^) ^a^	Onset Potential (mV)	j_f_ (mA mg^−1^_Pt_)	j_spec_ (mA cm^−2^) ^a^
Pt/C	−336	1015 at 116 mV	2.38	−282	1221 at 233 mV	2.86
Pt-Sn_1:1_	−379	1486 at 65 mV	2.44	−285	2583 at 296 mV	4.24
Pt-Sn_2:1_	−380	1294 at 111 mV	3.22	−285	1493 at 276 mV	3.71
Pt-Sn_3:1_	−337	993 at 145 mV	1.89	−285	1629 at 211 mV	3.09
Pt-Ru_1:1_	−539	1175 at 48 mV	1.45	−428	1803 at 103 mV	2.22
Pt-Ru_2:1_	−489	1248 at 83 mV	2.35	−402	1876 at 200 mV	3.53
Pt-Ru_3:1_	−514	1858 at 121 mV	2.49	−440	2434 at 199 mV	3.26

All potentials are referred to SHE. ^a^ Specific activity, j_spec_ = j_f_/ECSA, normalized to the real Pt surface area and expressed in mA cm^−2^.

**Table 6:** Chronoamperometric parameters of the EOR and EGOR: initial current density (I_s_) and final current density (I_f_) after 30 min at −100 mV vs. SHE at Pt/C, Pt-Sn/C, and Pt-Ru/C catalysts series in alkaline media.

Sample	EOR		EGOR	
	I_s_ (mA mg^−1^_Pt_)	I_f_ (mA mg^−1^_Pt_)	I_s_ (mA mg^−1^_Pt_)	I_f_ (mA mg^−1^_Pt_)
Pt/C	1130.95	30.38	1007.41	765.28
Pt-Sn_1:1_	1705.79	35.99	2085.61	1496.33
Pt-Sn_2:1_	1451.06	14.03	1308.06	891.43
Pt-Sn_3:1_	1102.87	13.49	1415.77	1023.65
Pt-Ru_1:1_	1265.34	0.71	2028.03	1188.99
Pt-Ru_2:1_	1319.29	1.69	1748.79	959.71
Pt-Ru_3:1_	1840.49	7.76	2352.04	1663.40

**Table 7:** CO adsorption energies (E_ads_, eV) on different surface sites (111) of Pt_sim_, Pt-Sn and Pt-Ru simulated model surfaces.

Sample	Pt_Atop_	M_Atop_	Bridged	Hollow
Pt_sim_	−1.735	-	−1.636	−1.876
Pt-Sn_1:1sim_	−1.133	0.016	−0.844	−0.052
Pt-Sn_3:1sim_	−2.320	−0.916 ^b^	−0.924 ^b^	−2.329 ^a^
Pt-Ru_1:1sim_	−1.374	−2.131	−1.990	−1.985
Pt-Ru_3:1sim_	−1.582	−2.179	−2.090	−1.507

^a^ The CO molecule initially placed at the hollow site migrated during relaxation to a Pt atop configuration; the reported value corresponds to the final Pt_Atop_ geometry. ^b^ The CO molecule did not remain chemisorbed at these sites; in the final geometries no Sn-C bond is formed and the reported values correspond to the resulting unbound configurations near the surface.

**Table 8:** d-band center of the surface Pt atom (ε_d_) and corresponding CO adsorption energy at Pt_Atop_ site (E_ads_) for Pt_sim_, Pt-Sn and Pt-Ru simulated model surfaces. Δ values in eV are referenced to Pt_sim_.

Sample	ε_d_	Δε_d_	E_ads_ (CO/Pt_Atop_)	ΔE_ads_
Pt_sim_	−2.013	-	−1.735	-
Pt-Sn_1:1sim_	−2.405	−0.392	−1.133	0.602
Pt-Sn_3:1sim_	−2.078	−0.065	−2.320	−0.585
Pt-Ru_1:1sim_	−3.233	−1.220	−1.374	0.361
Pt-Ru_3:1sim_	−2.318	−0.305	−1.582	0.153

## Data Availability

The original contributions presented in this study are included in the article. Further inquiries can be directed to the corresponding author.
